# Synthetic Biology Speeds Up Drug Target Discovery

**DOI:** 10.3389/fphar.2020.00119

**Published:** 2020-02-26

**Authors:** Yixuan Xie, Yanfang Yang, Yu He, Xixi Wang, Peng Zhang, Haocheng Li, Shufang Liang

**Affiliations:** ^1^ State Key Laboratory of Biotherapy and Cancer Center, West China Hospital, Sichuan University, and Collaborative Innovation Center for Biotherapy, Chengdu, China; ^2^ Department of Urinary Surgery, West China Hospital, West China Medical School, Sichuan University, Chengdu, China; ^3^ Department of Mathematics and Statistics, University of Calgary, Calgary, AB, Canada

**Keywords:** synthetic biology, gene circuit, logic gate, drug target, CRISPR-Cas9, functional screening

## Abstract

As a rising emerging field, synthetic biology intends to realize precise regulations of cellular network by constructing artificial synthetic circuits, and it brings great opportunities to treat diseases and discover novel drug targets. Depending on the combination mode of different logic gates, various synthetic circuits are created to carry out multilevel regulations. In given synthetic circuits, drugs often act as inputs to drive circuits operation. It is becoming available to construct drug-responsive gene circuits for experimentally treating various disease models, including metabolic disease, immunity disease, cancer and bacterial infection. Synthetic biology works well in association with the CRISPR system for drug target functional screening. Remarkably, more and more well-designed circuits are developed to discover novel drug targets and precisely regulate drug therapy for diseases.

## Introduction

In the last few decades, the inherent characteristics of biological components have inspired biologists to research deeply, and some of them have been identified as drug targets. Previous researches often focus on single level of biological regulation while ignoring the temporal and spatial properties of physiological processes. Current medicine requires precise drug therapy for regulation of dynamic pathological state, hence the synthetic biology emerges at the right time.

Getting inspiration from structural engineering, synthetic biology is an application-driven discipline to design and create standard, decoupling and abstracting biological components for engineering applications which do not exist naturally before (Endy, 2005; Way et al., 2014; Xie and Fussenegger, 2018; Wu et al., 2019). It aims to build artificial cellular networks, perform user-defined functions and finally generate oriented engineered cells or organisms (Saltepe et al., 2018; Sedlmayer et al., 2018; Wu et al., 2019). Generally, basic biological components make up biological devices, and then several biological devices form complex biological systems. From biological components to biological devices to biological systems, it reflects on the hierarchical composition of synthetic biology (Purnick and Weiss, 2009).

At the early stage of synthetic biology, optimizing or exploring of natural products by reconstructing microorganism metabolisms attracts much attention (Luo et al., 2014). As the advanced technology progresses, the synthetic biology-based therapeutic potential is becoming more commonly applied in mammalian disease treatment. With the idea of gene and engineered-cell therapies being put forward, the personalized medicine becomes prevalent gradually (Kitada et al., 2018). Drug target discovery has been of great importance for development of novel drugs and therapeutics. Traditionally, drug target screening and validation are usually dependent on chemical probes, which requires high selectivity in complex cellular system. And the synthesis of chemical probes probably generates false positive results due to change of molecular conformation. The chemical probes sometimes affect bioactivity of small molecule drugs and then cause wrong judgement. By contrast, synthetic biology-driven drug target discovery depends on the response of intracellular dynamic regulation and the phenotypic change without modifying drugs, which is a more realistic way to reflect existence of targets. In short, synthetic biology represents a huge potential to discover novel drug target and design new treatment strategy for diseases.

## Conventional Structure of Conceptual Synthetic Circuits

**Figure 1:**
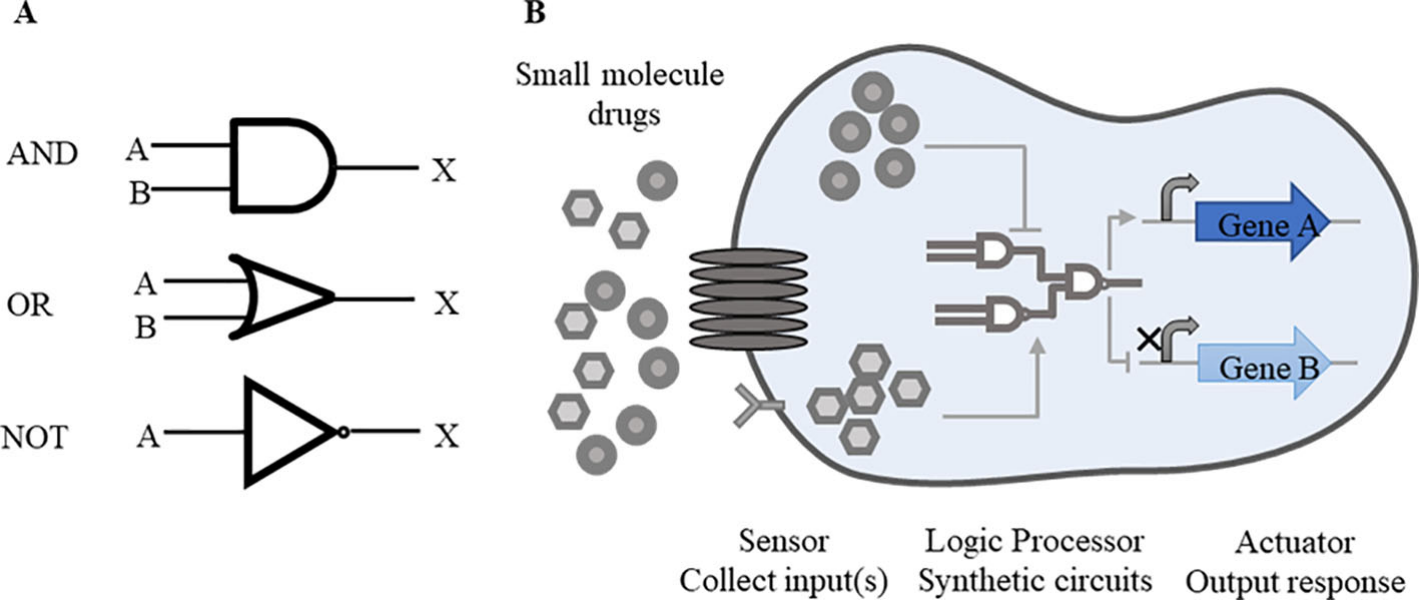
Basic synthetic circuits working pattern. **(A)** Common logic gates. “AND”, “OR” and “NOT” logic gates are commonly used forms in synthetic circuits. A, B represent inputs and X represents output. Simple symbols are used to express the logical relations between inputs and outputs. In “AND” gate, both inputs A and B are required for the output of X. In “OR” gate, either A or B is required for the output. In “NOT” gate, once the input A is working, the output X is suppressed. These logic gates function alone or in combination according to the level of regulation. **(B)** Drug-responsive synthetic circuits. In drug-responsive synthetic circuits, drugs often perform as inputs to initiate whole circuits. When drugs enter inside cells by going cross cell membrane or combining with receptors on the surface of membrane, logic gates response to drugs, following the transcription of target genes and other essential genes will be activated or repressed consequently.

Rational designment of synthetic circuits is one key issue of synthetic biology. A general synthetic circuit mainly consists of three components, including a sensor that collects input(s) from the internal and external, a logic processor responsive to inputs, and an actuator that outputs the expected response (Wu et al., 2019). These constituent parts, like as components of a machine, are designed independently before being integrated together to a man-made circuit. Synthetic circuits consist of several modularized elements, such as switches (Matsuura et al., 2018), oscillators (Riglar et al., 2019), cascades (Pickar-Oliver et al., 2019), feedback loops (Aoki et al., 2019), and Boolean logic gates (Green et al., 2017; Mircetic et al., 2017; Xia et al., 2019). Among these elements, Boolean logic gates are most commonly used forms (Saltepe et al., 2018). In order to facilitate the understanding of synthetic circuits, these can be abstracted as the form of logic gates. Similar with digital circuits, “AND”, “OR,” and “NOT” logic gates are widely applied in the design of synthetic biological circuits and switches (**Figure 1A**).

Other more complex circuits are established on the basis of these simple logic gates with multiple inputs and multiple outputs. For example, “NAND” gate is composed with a “NOT” gate and a “AND” gate. To achieve the expected outputs, “AND” gate requires two inputs present at once, while one feasible input is needed in “OR” gate. “NOT” gate shows the reverse trend between the inputs and the outputs. Through the setting of these logic gates, precise links are established between the input(s) and the output(s).

The synthetic circuits based on DNA, RNA or protein are designed to modulate endogenous cellular networks by precisely controlling expression of these biological molecules (Kitada et al., 2018). Through these biological modules, multiple levels of regulation will meet expectations clearly. Genetic devices made of single or multiple inputs/outputs are available to probe cellular action mechanisms (**Figure 1B**). In drug-responsive circuits, small molecule drugs often serve as inputs and target loop components, so as to start up or suppress gene expression.

## Small Molecule Drug-Responsive Synthetic Circuits Applied in Disease Models

Small molecule drugs often function as inputs to drive the synthetic circuits. Several small molecule drugs-involved gene circuits have been attempted to experimentally treat various diseases, including bacterial infectious diseases, immunity diseases, metabolic diseases and cancers (**Table 1**). Based on the binding of drugs and their corresponding targets, the artificial circuits are able to be pushed forward to activate or repress the downstream signaling pathways which exist in cellular environment or the well-designed circuits.

**Table 1:** Small molecule compounds-responsive synthetic circuits.

Disease classification	Small molecule compounds	Synthetic devices	Regulating elements	Output effects	Cell lines	Refs
Bacterial infectious diseases	2-phenylethyl-butyrate	DNA	EthR	Increasing sensitivity to drug	HEK-293 cells	Weber et al., 2008
Immunity diseases	Leucovorin (6R)- folinic acid	DNA	miRNA	Modulating T cell proliferation	T cells	Wong et al., 2018
	Doxycycline	DNA	Tetracycline inducible promoter (*pTRE*)	Disabling T cell activation temporarily	T cells	Wei et al., 2012
	Theophylline	RNA	Ribozyme	Controlling cell proliferation	mouse and primary human T cells	Chen et al., 2010
Metabolic diseases	Guanabenz	DNA	Chimeric trace amine-associated receptor	Stimulating the secretion of active peptides	HEK-293 cells, Hela, Hana3A cells and CHO-K1	Ye et al., 2013
	Cytosine arabinoside (Ara-C)	DNA	The *luxCDABE* operon	Detecting the transition between Ara-C and Ara-CTP	*E. coli* MG1655	Alloush et al., 2010
	Phloretin	DNA	Bacterial DNA-binding repressor TtgR	Inhibiting the downstream transgene expression	HEK-293 cells, BHK-21, COS-7, CHO-K1, Hela, HT-1080 and human mesenchymal stem cells	Rossger et al., 2013
	Protocatechuic acid (PCA)	DNA	KRAB-PcaV transrepressor fusion protein	Increasing the insulin level and lowering the blood glucose concentrations	HEK-293 cells, HeLa, human telomerase-immortalised mesenchymal stem cells, mouse myoblast cells (C2C12), and HEK-293-derived Hana3A cells	Yin et al., 2019
Cancers	Ganciclovir	DNA	Herpes simplex virus-thymidine kinase	Inducing cell apoptosis	HEK-293 cells	Culler et al., 2010
	4-hydroxytamoxifen (4-OHT)	DNA	The estrogen receptor ligand binding domain (ERT2)	Controlling CAR expression and T cell activity	Jurkat T cells	Chakravarti et al., 2019
	4-hydroxytamoxifen (4-OHT)	DNA	ERT2-CreN-nMag	Controlling CAR expression and T cell activity	HEK293T cells, Jurkat T cells (Clone E6-1, TIB-152), K-562 lymphoblasts (CCL-243, CD38-/CD19- target cells), and Toledo B lymphocytes	Allen et al., 2019
	Doxycycline, Trimethoprim	RNA	Tetracycline-responsive repressor and *E. coli* dihydrofolate reductase	Controlling the expression of fusion proteins	BHK-21 cells and C2C12 mouse myoblasts	Wagner et al., 2018
	Theophylline	RNA	Ribozyme	Causing cell cycle arrest	U2-OS cells and HEK-293 cells	Wei and Smolke, 2015

The regulation elements in various disease models include kinases, promoters, activators and repressors. Drug sensitivity is increased by designing synthetic circuits to kill pathogenic bacteria. Just as a typical example in *Mycobacterium tuberculosis*, the repressor of *ethA* (EthR) binds to a specific operator to inhibit the ethionamide monooxygenase (EthA), which catalyzes conversion of the prodrug ethionamide to an antimycobacterial nicotinamide adenine dinucleotide derivative (Weber et al., 2008). Based on repression of the binding between EthR and the promoter by 2-phenylethyl-butyrate, a synthetic circuit is designed to sense the EthR-operator interaction in human HEK-293 to control EthA enzyme activity for prodrug biochemical conversion (Weber et al., 2008).

Synthetic biology ideas are applicable for intervening immunity therapy. The leucovorin-mediated microRNA switches are used to modulate T cell proliferation by targeting the endogenous cytokine receptor subunits (Wong et al., 2018). Other study reports T cell activation can be temporarily disabled through a pause switch inducing by doxycycline (Wei et al., 2012). In T cells, a transient receptor of the potential melastatin 8 channel is activated by adding menthol to increase intercellular calcium, which induces calcium-responsive nuclear factors of activated T cells to translocate and bind to specific promoters to stimulate expression of secreted alkaline phosphatase (Bai et al., 2019).

Synthetic circuits for regulating metabolic diseases are in progress. For example, cytosine arabinoside (Ara-C) is a key agent for treating acute myeloid leukemia by converting cytosine arabinoside triphosphate (Ara-CTP) for functions. A cytidine deaminase-deficient *E. coli* mutant MG1655 contains *luxCDABE* genes encoding luciferase, which is responsive to Ara-C stimulation (Alloush et al., 2010). In acute myeloid leukemia cells, Ara-CTP converts to Ara-C in the absence of cellular alkaline phosphatase, then Ara-C enters into cells to open the luminous gene “ON” to reflect drug sensitivity in acute myeloid leukemia cells. By designing luminous circuit, the transition efficiency between Ara-C and Ara-CTP is detectable in patients’ leukemic cells (Alloush et al., 2010). Beyond that, a synthetic signal cascade is activated through inputting Guanabenz, a common antihypertensive drug. Guanabenz is identified to activate chimeric trace amine-associated receptor 1, and finally stimulates the secretion of active peptides GLP-1 and leptin to therapy metabolic syndrome (Ye et al., 2013). By designing “AND” gate, fatty acids and phloretin perform as dual inputs in an intracellular lipid-sensing receptor. Under the condition of absence of fatty acids, phloretin binds to bacterial DNA-binding repressor of *ttg* genes (TtgR), the expression of downstream transgene will be inhibited (Rossger et al., 2013). Lately a switch induced by protocatechuic acid (PCA) showed splendid treatment effect in type 1 and type 2 diabetes. The systems which contain transcriptional repressor PcaV can boost the level of insulin and reduce blood glucose concentrations in diabetic mice and monkeys (Yin et al., 2019).

Moreover, several DNA systems bring great hope for therapy of tumor diseases through precisely acting on drug target. Typical case is using ganciclovir to control cell survival. Under the β-catenin and NF-κB pathway stimulation, the exons before herpes simplex virus-thymidine kinase, which is sensitive to ganciclovir, are repressed then the output of herpes simplex virus-thymidine kinase induce cell apoptosis (Culler et al., 2010). Recently, the chimeric antigen receptor (CAR) T-cell immunotherapy becomes popular because of its accuracy and individuation. Different synthetic circuits are designed to modulate the status of T cells. For example, DNA circuits control CAR expression and T cell activity on the inducible condition of 4-hydroxytamoxifen (Chakravarti et al., 2019). Another novel AND gate system named TamPA-Cre system, which includes inducible Magnet protein domains (nMag, pMag) and split Cre recombinase, can realize localized CAR expression by using 4-hydroxytamoxifen and blue light successively and thus control T cell activation in solid tumor (Allen et al., 2019).

Besides DNA gene circuits, RNA-based circuits also perform well. By using modified messenger RNAs or riboswitches, random genomic integration can be limited than using DNA delivery system so that it may be a safer way to conduct functions. For example, applying doxycycline and trimethoprim, TetR-DDX6 fusion proteins control expression of proteins from RNA-encoded genetic circuits (Wagner et al., 2018). Synthetic circuits responsive to theophylline depend on ribozyme switch and regulate the expression of CCNB1m, which causes U2OS cell cycle arrest in the G0/1 or G2/M phases (Wei and Smolke, 2015). Also applying theophylline to T cells, another RNA-based device controls cell proliferation through regulating IL-2 (Chen et al., 2010).

## Synthetic CRISPR-Cas System Improves High-Throughput Identification of Drug Targets

### Brief Introduction of Several CRISPR-Cas Systems

The essentiality of cellular function is possibly related with the degree of evolvability, so genes with the least evolvability (essential genes) have maximum essentiality and may be the better candidates for drug targets (Rancati et al., 2018). Essential genes also tend to encode proteins that engage in more protein-protein interactions and participate in multiple regulations (Wang et al., 2015). Therefore, finding genes with the least evolvability is the primary task for identifying drug targets.

Molecular biology serves as a powerful tool to turn genes on and off. The principle difference between molecular biology and synthetic biology is that synthetic biology assembles parts from molecular biology (Macdonald and Deans, 2016). As the most well-known system of synthetic biology, CRISPR-Cas9 system is a convenient tool for site-directed mutation and identification of gene function. Cas9 is a member of Cas endonucleases. Among these endonucleases, the most famous and well-studied are Cas9, Cas12a (previously known as Cpf1), Cas13a, and Cas13b (Zetsche et al., 2015). Both Cas9 and Cas12a are targeting DNA, while Cas13a and Cas13b are targeting RNA.

Compared with Cas9, Cas12a recognizes G-rich protospacer adjacent motif (PAM) while Cas9 recognizes T-rich PAM, which is complementary to Cas9 system and enlarges the range of recognition (Zetsche et al., 2015). Further, Cas12a is guided by CRISPR RNAs which has less length than Cas9, and it is facilitate to multiple genetic manipulation and packaging into viral vectors (Tang et al., 2017). Despite these advantages above, CRISPR-Cas9 system has much wider range of use than CRISPR-Cas12a system because of the difficulties to modify Cas12a using the similar way as for Cas9 based on the difference of their structures (Yamano et al., 2017). On the bright side, several efforts were made to improve CRISPR-Cas12a system. For example, an optimizing CRISPR‐Cas12a system realized seamless DNA editing in one pot (Wang et al., 2019). And a pair of split Cas12a and deficient/dead Cas12a (dCas12a) fragments showed potent efficiency in both rapamycin-inducible, photoactivatable genome editing and endogenous gene activation (Nihongaki et al., 2019). Recently Cas13 systems are further explored to more precisely cleave virus RNA (Freije et al., 2019) and track RNA dynamically (Yang et al., 2019). It considers a desirable system to become more productive in drug target discovery.

Here, several novel techniques derived from CRISPR-Cas system are carrying out in drug targets screening and identification for mammalian disease treatment.

### CRISPR-Based Systems for Functional Gene Screening

**Figure 2:**
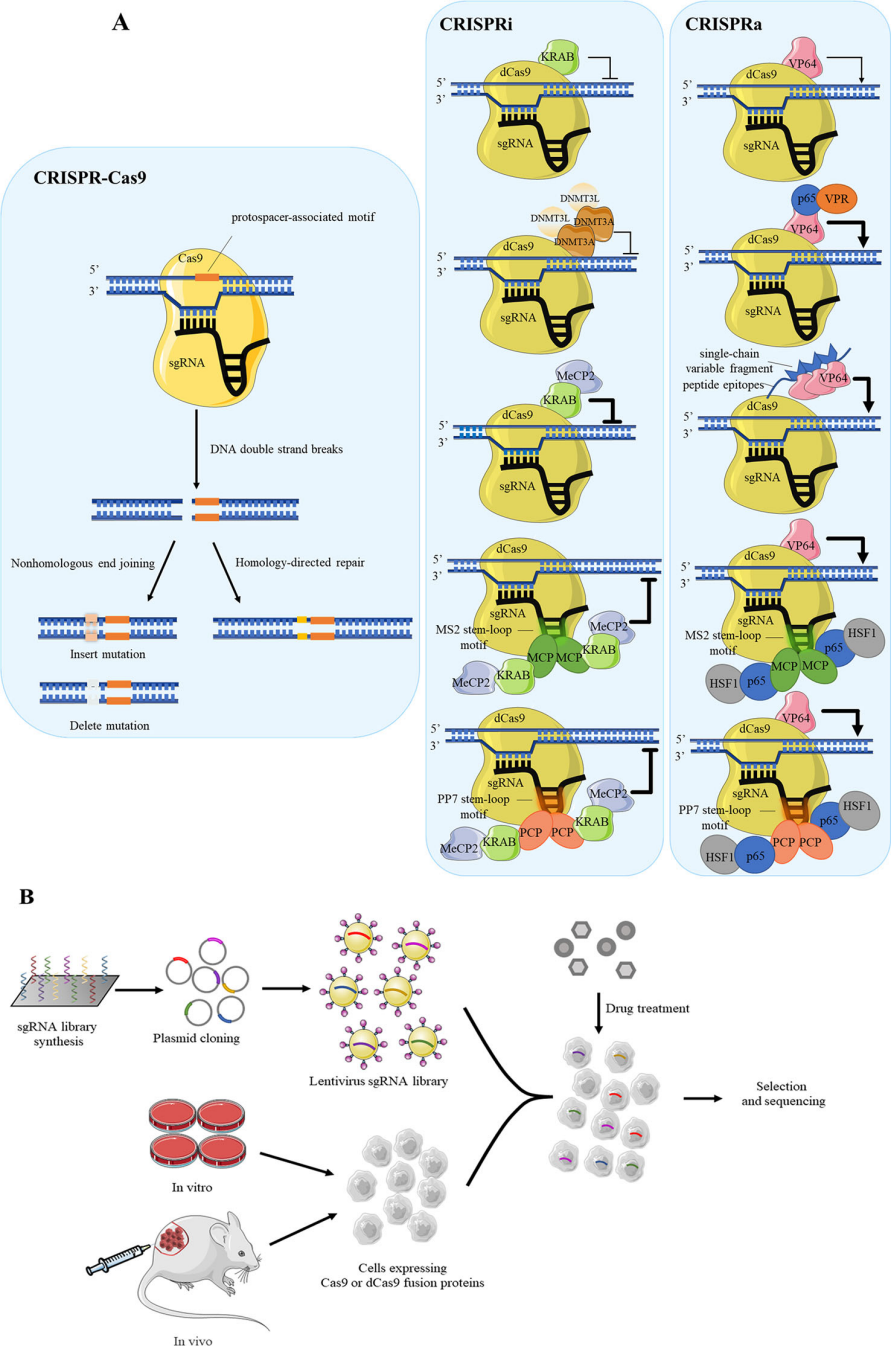
Several CRISPR systems are applied for drug target screening. **(A)** The schematic diagram of CRISPR-Cas9 system, CRISPRi, CRISPRa and their variants. The CRISPRi and CRISPRa distinguish CRISPR/Cas9 system with the dCas9 rather than Cas9. Cas9 combined with sgRNA perform shearing function to specific site on target DNA, causing DNA double-strand breaks. The gene repair approaches include the nonhomologous end joining and homology-directed repair. For achieving higher efficiency, dCas9 often fuses with repressed proteins such as KRAB and DNMT3A in CRISPRi, while in CRISPRa it often fuses with activated protein VP64. **(B)** The flow diagram of cell-based high-throughput screening using pooled sgRNA library synthesis. The synthesized sgRNAs are cloned into plasmid for amplifying by lentivirus to establish sgRNA library. Cells which are expressed Cas9 or dCas9 undergo drug treatment to select against sgRNA library according to phenotype changes, following the drug target genes are analyzed by the next generation sequencing (NGS).

CRISPR-Cas9 is a currently popular tool for purpose of functional gene screening and validation due to its high efficiency with minimal off-target effect than RNA interference. The Cas9 cleaves specific genomic loci which includes the protospacer-associated motif to form DNA double-strand breaks, following Cas9-mediated genome editing by the nonhomologous end joining or homology-directed repair in mammalian cells (Doudna and Charpentier, 2014; Hsu et al., 2014) (**Figure 2A**).

Recently, several CRISPR-modified vectors are developed to satisfy the need of gene function research, and one of typical example is CRISPR interference (CRISPRi). Different from routine CRISPR-Cas9 system, CRISPRi is mediated by dCas9 protein (Qi et al., 2013), which is disable to mediate DNA double-strand breaks while reserving the ability of RNA-guided genomic targeting. To enhance the repressive capacity, dCas9 is often fused with effectors such as the transcription repression domain of Krüppel-associated box (KRAB) (Gilbert et al., 2013). Another similar example is DNMT3A as repressor to cause DNA methylation to silence downstream gene (Vojta et al., 2016). Specially, DNMT3A induces DNA methylation as the form of dimer, and usually recruits its partner DNMT3L. Recently, Yeo et al. has established an improved method using dCas9-KRAB-MeCP2 fusion protein for better efficiency (Yeo et al., 2018). The function of MeCP2 is consistent with DNMT3A, which binds with methylated DNA for repression. Using the dCas9 fusion protein guided by gene-specific single guide RNA (sgRNA), the effector domains localize in specific DNA sequences, such as promoters, 5’ untranslated regions or enhancers (Gilbert et al., 2014; Pickar-Oliver and Gersbach, 2019). The dCas9 protein fused to transcription repression domain corresponds to “NOT” gate, hence the expressions of downstream target genes are “OFF.” For example, CRISPR/dCas9-KRAB inhibits the expression of individual host factors, RIG-I and PKR, which are considered to influence the inhibitory effect of nitazoxanide against Ebola Virus (Jasenosky et al., 2019).

On the other hand, for gene activation, CRISPR activation (CRISPRa) is developed to assess the phenotypic changes derived from overexpressed genes (Gilbert et al., 2014; Konermann et al., 2015). dCas9 protein which often binds to transcriptional activation domains has a tendency to act as the “ON” switch, consequently the target genes are activated. VP64, which contains a transcriptional activator domain, binds to dCas9 for activation function (Mali et al., 2013) (**Figure 2A**). To date, researchers have designed variants on the basis of dCas9-VP64 system with higher efficiency. For instance, Chavez et al. constructed a tripartite activator which contains VP64, P65 and Epstein–Barr virus R transactivator Rta (Chavez et al., 2015). Similarly, a synergistic activation mediator is made of three parts, P65, HSF1 and MS2 bacteriophage coat protein (Konermann et al., 2015). In addition to the case above, a protein scaffold named SunTag is designed to recruit many copies transcriptional activation domain, such as VP64, to dCas9 protein. This scaffold shows much higher efficiency than plain system because it recruits multiple proteins at once and amplifies the activation effect (Tanenbaum et al., 2014). Other scaffolds are made of RNA stem-loop motif, MS2 and PP7 (Konermann et al., 2015; Fu et al., 2016). MS2 and PP7 are viral RNAs which respectively recruit the bacteriophage coat proteins MS2 coat protein (MCP) and PP7 coat protein (PCP) to the RNA hairpins. The typical activation mediator is made of three parts, P65, HSF1 and bacteriophage coat proteins. This system can not only be used in CRISPRa, but also work well in CRISPRi, which needs replace P65-HSF1 fusion protein with KRAB-MeCP2 fusion protein (Martella et al., 2019).

Besides CRISPR-Cas9 system, base editor is another powerful technology to explore gene function. This approach does not result in DNA cleavage, which is considered as a safer tool than Cas9 system (Kim et al., 2017). Previous base editors, including cytidine base editors (Kim et al., 2017) and adenine base editors (Gaudelli et al., 2017) which mainly consist of catalytically deficient/dead Cas9 (dCas9) protein and cytidine deaminase or adenosine deaminase, realize C to T (or G to A) base transition in mammalian cells. The well-modified dCas9-AIDx system induces specific gene mutation to screen imatinib-resistance tumor cells, thus identifying mutation sites of drug-resistance related gene (Ma et al., 2016). Latest base editors named Prime Editor bring about transversion mutations by precise genome editing with higher product purity and efficiency (Anzalone et al., 2019). In spite of its convenience, the off-target effects cannot be ignored (Zuo et al., 2019) and the application potential remains to be further explored.

In eukaryotes, the activation of endogenous genes results in cellular reprogramming which is linked with the phenotypic changes (Black and Gersbach, 2018). Furthermore, not only designing individual sgRNA which targets one gene, Farzadfard et al. designed plasmids encoding three layers of orthogonal sgRNAs to control cell activity precisely. Only when cognate interactions between sgRNAs and target binding sites exist can HEK293T cells achieve maximum activation (Farzadfard et al., 2013).

### CRISPR Screening Application in Cells

Drawing inspiration from the design of layers gRNAs, it is evident that CRISPR-Cas9 system has huge potential to regulate multilevel transcriptional networks and conduct high-throughput screening. Aiming at screening of culture cells, sgRNA libraries are necessary. There are two general ways to generate library, arrayed or pooled (Shalem et al., 2015). The pooled is the more commonly used format because of the availability of oligonucleotide library synthesis technologies. In pooled formats, large numbers of sgRNA hairpins are synthesized on oligonucleotide arrays and often cloned into lentiviral vectors. The library is packaged into lentiviruses and used to transduce cells at a low multiplicity of infection so that most cells receive only one hairpin (Luo, 2016). Comparison of cell response to drug interference is an available strategy in drug target identification. Then, the expressing gene differences between untreated and treated population are analyzed by the next generation sequencing. Through quantifying the readout, the composition of libraries and sgRNA abundance between samples can be identified (**Figure 2B**).

It usually works well to build a CRISPR-Cas9 knockout library for CRISPR screen. CRISPR-Cas system speeds up drug target screening owing to its high efficiency. Small molecule drugs act as inputs to screen target genes. For example, the connect between drug metabolism and the effect of drug on gene expression are realized in acetaminophen induced hepatotoxicity (Shortt et al., 2019). The ICAP12 gene is discovered to be essential for the fitness of parasites through CRISPR screen approach in combination with the antiparasitic compound 5-fluorodeoxyuridine to study apicomplexan parasites causing malaria and toxoplasmosis (Sidik et al., 2016).

The generation of drug resistance brings a huge challenge for disease drug treatment, which is triggered by several aspects including drug-resistance genes (Holohan et al., 2013). So far, identification of drug-resistance genes is helpful for exploring drug resistance molecular mechanisms and precision drug administration for patients. Ophir Shalem et al. screened drug resistance genes against vemurafenib (RAF inhibitor) in a melanoma model (Shalem et al., 2014). In imatinib-resistant gastrointestinal stromal tumor, nine genes (DBP, NR3C1, TCF12, TP53, ZNF12, SOCS6, ZFP36, ACYP1, and DRD1) are identified as novel targets (Cao et al., 2018). Target genes involved in the DNA mismatch repair pathway are identified for resistance to the nucleotide analog 6-thioguanine (Wang et al., 2014).

Besides CRISPR-Cas9 screen, CRISPRi/a screen is also wildly applied for target gene discovery. Distinct from CRISPR screen, dCas9 (fusion) proteins replace Cas9 proteins to function in the latter system. CRISPRi screen is helpful for discovery of drug resistance gene. In TP53 wild-type Ewing sarcoma, genes MDM2, MDM4, USP7, and PPM1D have been identified to be responsible for druggable dependencies through CRISPRi screen of three inhibitors to compare cell responses to drug treatment (Stolte et al., 2018). Combining CRISPRi and CRISPRa screens, phenotypes of rigosertib sensitivity determined by many genes are detected simultaneously (Jost et al., 2017). It is defined that the mutation of microtubules is combined with rigosertib’s binding pocket from the point of structural biology. Similarly, cell sensitivity to a chimeric cholera/diphtheria fusion toxin reveals the well-known and unknown mechanisms in sphingolipid metabolism and diphthamide biosynthetic pathway (Gilbert et al., 2014).

### CRISPR Screening in Animal Disease Models

In addition to apply in cell lines, CRISPR screening is also expanded to animal models for identifying genes associated with tumor growth or evaluating drug responses. After the engineering cells carried with sgRNA libraries are constructed, these cells are injected into the immunodeficient mice (Katigbak et al., 2016) or xenograft mice (Shalem et al., 2014; Manguso et al., 2017) through hydrodynamic injection or intraperitoneal injection. After treatment, similar with *in vitro* strategy, the cells which are isolated from the tumor tissues are send to go through next generation sequencing.

Of course, specific genes sensitive or resistant to immunotherapy are efficiently identified through CRISPR-Cas9 screen on mouse models. Manguso et al. transplanted B16 melanoma cells, which include Cas9 and sgRNA library, into T cell deficient mice to identify target genes responsive to immunotherapy (Manguso et al., 2017). After observations of treatment with tumor cell vaccine (GVAX) or GVAX combined with anti-PD-1 into mice, it is confirmed that IFN-γ signaling genes cause drug resistance. On the contrary, genes in NF-κB signaling, antigen presentation and the unfolded protein response pathways are sensitive to immunotherapy (Manguso et al., 2017).

## Conclusions and Perspectives

Synthetic biology provides new ideas and ways for drug target discovery. However, there are several challenges that cannot be ignored. Most gene circuits and elements are artificial, and these components do not exist in human body intrinsically. Thus the immune responses caused by these exogenous components are hard to evaluate accurately (Wei et al., 2016; Charlesworth et al., 2019). Possible solutions are to develop low immunogenic systems (Moreno et al., 2019) to have favorable biocompatibility by implanting encapsulated cells carrying synthetic circuits with biomaterials (Lathuiliere et al., 2014; Ye et al., 2017). Furthermore, as a genome-engineering tool, CRISPR-Cas9 system probably generates off-target effects at amplified loci (Munoz et al., 2016) and DNA damage is induced by Cas9. Therefore, more precise circuits should be developed to cause lower endogenous noise and achieve targeted cell therapy or drug treatment. For example, blood glucose of diabetic mice and monkeys is subtly controlled by a synthetic switch that is triggered by a green tea compound (protocatechuic acid) with rapid absorption rate and no burden on organisms (Yin et al., 2019).

In addition, drug combination therapy is an efficient method for clinical disease treatment. Multidrug-resistance is more complex than single drug-resistance, and the alterations of drug targets may take place in spatial and temporal levels. All of these factors bring challenges to drug targets identification. A recent report created a novel system using 4-hydroxytamoxifen and blue light as external inputs, which can realize spatiotemporal control of CAR-T cell activity in solid tumor (Allen et al., 2019). Aiming at personalized medicine, it remains a puzzle that how to control the dose and time of using multidrug. Directing at multidrug screening, layering screening circuits will be needed and more complex algorithms are promising to add in.

## Author Contributions

Wrote or contributed to the writing of the manuscript: YX, YY, YH, XW, PZ, HL and SL. All authors approved the paper for publication.

## Funding

This work was supported by National Natural Sciences Foundation of China (31961143005) and Chengdu Science & Technology Program (2017-GH02-00062-HZ).

## Conflict of Interest

The authors declare that the research was conducted in the absence of any commercial or financial relationships that could be construed as a potential conflict of interest.

## Abbreviations

Ara-C, cytosine arabinoside; Ara-CTP, cytosine arabinoside triphosphate; CAR, chimeric antigen receptor; PAM, protospacer adjacent motif; dCas12a, deficient/dead Cas12a; dCas9, deficient/dead Cas9; CRISPRi, CRISPR interference; KRAB, Krüppel-associated box; sgRNA, single guide RNA; CRISPRa, CRISPR activation.
